# Catheter-Based Regional Anesthetic Techniques for Comprehensive Pain Management and Early Mobilization After Cardiac Sternotomy: A Report of Two Cases

**DOI:** 10.7759/cureus.81721

**Published:** 2025-04-04

**Authors:** Keisuke Nakazawa, Osamu Kitajima, Takahiro Suzuki

**Affiliations:** 1 Anesthesiology, Nihon University School of Medicine, Tokyo, JPN

**Keywords:** cardiac surgery, catheter technique, deep parasternal intercostal plane block, early mobilization, intermittent bolus, multimodal analgesia, poststernotomy pain, rectointercostal fascial plane block, regional anesthesia, superficial parasternal intercostal plane block

## Abstract

Post-sternotomy pain management following cardiac surgery remains challenging, with both sternal incision and drain site pain requiring effective control. As highlighted recently, regional anesthetic techniques targeting the anterior cutaneous branches of the thoracic intercostal nerves offer promising solutions for comprehensive pain control while reducing opioid requirements. We present two patients who underwent cardiac surgery via sternotomy, with post-operative pain managed using different catheter-based regional anesthetic techniques. The first patient received bilateral deep parasternal intercostal plane block (DPIPB) catheters, while the second received a combination of a superficial parasternal intercostal plane block (SPIPB) and rectointercostal fascial plane block (RIFPB) with catheter placement. Both techniques demonstrated efficacy in managing post-sternotomy pain in our patients. The scheduled intermittent 0.25% levobupivacaine boluses provided analgesia that coincided with the patients' early mobilization activities, potentially contributing to their rehabilitation progress. The first patient achieved good pain control with DPIPB catheters when combined with oral analgesics, while the second patient, who received SPIPB as a single-shot block and RIFPB with indwelling catheters, achieved comprehensive pain control without requiring supplemental medications.

Both cases highlight the importance of addressing epigastric drain site pain, which often presents significant challenges in post-sternotomy pain management. These catheter-based techniques provide extended, adaptable analgesia during the critical early mobilization period after cardiac surgery, balancing effective pain control with safety considerations. Further research is needed to compare these different approaches systematically and identify optimal strategies for diverse cardiac surgical procedures.

## Introduction

Effective pain management following cardiac surgery via sternotomy is crucial for improving patient outcomes, facilitating early mobilization, and reducing respiratory complications. As highlighted in a recent practice advisory by the Society of Cardiovascular Anesthesiologists, moderate to severe pain after cardiac surgery is relatively common and increases the risk of post-operative cardiopulmonary complications while delaying hospital discharge [1]. While opioids have traditionally been used for post-cardiac surgery pain control, they are associated with serious adverse effects that can be particularly problematic in this patient population.

Post-sternotomy pain presents multiple challenges, with pain originating from both the sternal incision site and various drain sites requiring comprehensive control [2,3]. These areas are innervated primarily by the anterior cutaneous branches of the thoracic intercostal nerves (T2-T6 for the sternal region and T6-T8 for the lower chest and epigastric region), making targeted nerve blocks a rational approach for pain management.

Recent advances in ultrasound-guided regional anesthesia have led to the development of various fascial plane blocks targeting these nerves, including deep parasternal intercostal plane block (DPIPB) and superficial parasternal intercostal plane block (SPIPB) [2,4,5]. Cadaver studies [4,5] have demonstrated differences in dye spread patterns between these techniques, with DPIPB showing superior craniocaudal spread compared to SPIPB. However, despite these anatomical differences, both techniques have demonstrated comparable clinical efficacy in reducing opioid requirements after cardiac surgery, suggesting that the observed differences in dye distribution may not directly translate to clinically significant variations in analgesic effect.

Furthermore, the method of local anesthetic administration-whether as a single injection, continuous infusion, or intermittent boluses-may significantly influence the spread of local anesthetic and subsequent analgesic efficacy in these fascial plane blocks. Despite this potential impact, there is a notable scarcity of clinical studies specifically addressing how different administration techniques affect the outcomes of parasternal fascial plane blocks after cardiac surgery.

The advent of fascial plane blocks has significantly expanded regional analgesia options for cardiac surgical patients, previously limited by concerns about neurological complications with systemic anticoagulation [1]. While effective for sternal incision pain, comprehensive control throughout the surgical field often requires additional approaches. Catheter-based techniques for intermittent bolus administration extend the duration and adaptability of these blocks. Levobupivacaine, with its favorable safety profile and reduced cardiotoxicity, is particularly advantageous in cardiac patients [6]. The ability to administer additional doses during early mobilization may offer significant advantages over single-shot techniques, especially in patients with compromised cardiac function.

We present two cases demonstrating different catheter-based regional anesthetic approaches for post-sternotomy pain management, focusing on comprehensive analgesia during the critical early mobilization period.

## Case presentation

Both patients were managed under general anesthesia with standard cardiac monitoring. Anesthesia was induced and maintained with propofol (target-controlled infusion at 2-3 μg/mL), rocuronium (0.6 mg/kg for induction and 0.2 mg/kg as needed), fentanyl (total dose 200 μg), and remifentanil (0.1-0.3 μg/kg/min). Intraoperative analgesia was primarily maintained with continuous remifentanil infusion, supplemented with fentanyl (total dose 200 μg) and intravenous acetaminophen (1,000 mg) administered at the time of sternal closure to provide bridging analgesia. Post-operatively, our intensive care unit (ICU) pain management protocol included oral tramadol (37.5 mg)/acetaminophen (325 mg) combination tablets for patients reporting persistent pain scores > 5 on the Numerical Rating Scale (NRS) or expressing desire for additional analgesia. In cases where oral analgesics provided insufficient pain control, intravenous patient-controlled analgesia (IV-PCA) with fentanyl (10 μg/mL/hour, bolus 10 μg, lockout time 10 minutes) was available as a rescue option. The timing of intermittent boluses through the catheters was established at 12-hour intervals (morning and evening) based on our institutional standard protocol for fascial plane catheter management in cardiac surgical patients. This schedule was designed to coincide with nursing shift changes and planned mobilization activities, allowing for optimal pain control during physical therapy sessions. While more frequent administration could be considered based on individual patient needs and the pharmacokinetics of levobupivacaine, this standardized approach facilitated consistent care coordination across multiple providers while maintaining effective analgesia throughout the recovery period.

Prior to performing any regional anesthetic techniques, we confirmed that the activated clotting time (ACT) had normalized to <150 seconds following heparin reversal with protamine, in accordance with our institutional safety protocol for regional anesthesia in cardiac surgical patients. All nerve blocks were performed by a board-certified anesthesiologist who holds certification from the European Society of Regional Anaesthesia & Pain Therapy (ESRA), ensuring a high level of expertise in advanced regional techniques. The total procedure time, including preparation, antiseptic skin preparation, block performance, and catheter fixation, was approximately 10 minutes for both the bilateral DPIPB catheter placement in Case 1 and the combined SPIPB with RIFPB catheter placement in Case 2. For all nerve blocks, an 18G Contiplex® Touhy Ultra360 100 mm needle (B. Braun Melsungen AG, Melsungen, Germany) was used, and catheters were advanced 3 cm beyond the needle for post-operative analgesia. The nerve blocks were performed immediately after skin closure, before the patients were transferred to the ICU. Following standard post-operative cardiac monitoring, both patients were extubated in the ICU approximately 4-6 hours after surgery completion.

Case 1

**Figure 1:**
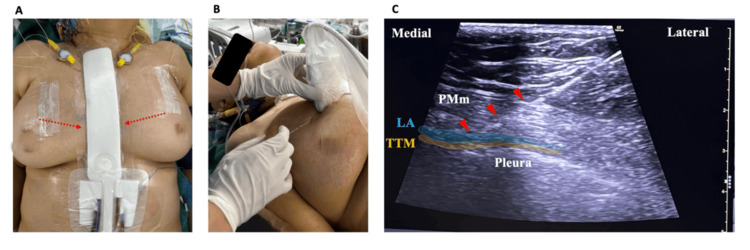
Deep parasternal intercostal plane block (DPIPB) procedure and catheter placement. (A) The red dotted arrows indicate the direction of catheter placement. (B) Linear ultrasound probe-guided placement and needle direction for DPIPB. (C) Ultrasound image showing the pectoralis major muscle (PMm), local anesthetic (LA), transversus thoracis muscle (TTM), and pleura. The red arrowheads indicate the needle trajectory.

A 68-year-old female patient (155 cm, 70 kg) underwent mitral valve repair for P3 prolapse. The operation duration was four hours and 20 minutes. Following sternotomy closure, bilateral DPIPB catheters were placed under ultrasound guidance at the T4-T5 intercostal level (Figure 1).

After confirming correct needle placement between the pectoralis major and transversus thoracis muscles, initial bilateral DPIPB was performed with 20 mL of 0.25% levobupivacaine on each side, followed by catheter insertion. Post-operatively, the catheters were maintained with intermittent boluses of 10 mL of 0.25% levobupivacaine every 12 hours on each side.

Post-extubation, the patient reported excellent sternal incision pain control (NRS score 0 at rest, 2 with coughing). Post-extubation cold testing (using an ice cube) demonstrated bilateral sensory blockade from T3 to T6 dermatomes. However, no blockade was observed in the anterior cutaneous branches of the intercostal nerves caudal to the costal margin, including the T7-T8 dermatomes where the drains were inserted.

On the post-operative day 1 (POD1) morning, the patient reported significant drain site pain in the epigastric region (NRS score 6 at rest and 10 with coughing), necessitating administration of 10 mL of 0.25% levobupivacaine through each DPIPB catheter. Cold testing at this time was negative, confirming the resolution of the sensory blockade. After the administration of 10 mL of 0.25% levobupivacaine through each DPIPB catheter, cold testing confirmed renewed sensory blockade in the T3-T6 dermatomes, but no blockade was achieved in the T7-T8 regions.

Despite the limited spread of local anesthetic to the epigastric region as confirmed by cold testing, a standardized regimen combining bilateral intermittent boluses administered twice daily through the catheters and oral tramadol (37.5 mg)/acetaminophen (325 mg) combination tablets twice daily successfully reduced pain to NRS scores below 2 at rest. This suggests that while the DPIPB catheters provided excellent sternal analgesia, the epigastric drain site pain was primarily managed through the addition of systemic analgesics. A standardized regimen was established with bilateral intermittent boluses administered twice daily (morning and evening) through the catheters, without continuous infusion. Although the patient experienced some breakthrough pain before the scheduled evening dose, this was adequately managed with the addition of oral analgesics. Oral tramadol (37.5 mg)/acetaminophen (325 mg) combination tablets were prescribed twice daily, which, along with the scheduled intermittent boluses through the DPIPB catheters, successfully reduced pain to NRS scores below 2 at rest.

The patient progressed to a sitting position on POD1 and standing at the bedside on POD2. By POD3, following drain removal, the patient was able to participate in walking rehabilitation with pain scores remaining below NRS 2. Given the successful pain management and rehabilitation progress, the DPIPB catheters were removed on POD3 after the pre-rehabilitation local anesthetic administration, with continued oral analgesics providing adequate pain control. Following catheter removal, no significant increase in pain intensity was observed, and the patient maintained NRS scores below 3 with oral analgesics switched to an as-needed basis rather than scheduled administration, without experiencing any rebound or sharp pain. By this point, the patient had already completed the most challenging aspects of early mobilization and the drains had been removed, which likely contributed to the reduced analgesic requirements.

Case 2

**Figure 2:**
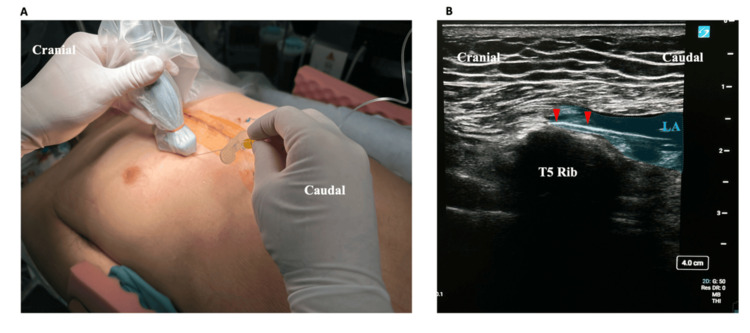
Superficial parasternal intercostal plane block (SPIPB) procedure. (A) Probe placement in the sagittal orientation. (B) Ultrasound image showing local anesthetic (LA) spread beneath the pectoralis major muscle (PMm). The block needle was advanced from the caudal to the cranial direction using the hydrodissection technique. The red arrowheads indicate the needle trajectory along the T5 Rib.

A 71-year-old male patient (167 cm, 76 kg) with three-vessel coronary artery disease and low ejection fraction (33%) underwent on-pump beating coronary artery bypass grafting (CABG). The operation lasted five hours and 10 minutes. Following surgery, SPIPB was performed using a two-stage hydrodissection technique with 20 mL of 0.25% levobupivacaine on each side (total 40 mL) bilaterally as a single-shot block (Figure 2).

The procedure was initiated at the T6 level with advancement to T4, followed by a second insertion from T4 with advancement to T2, allowing for comprehensive coverage of the parasternal region from T2 to T6.

**Figure 3:**
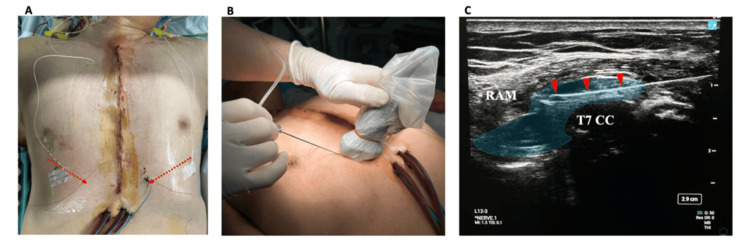
Rectointercostal Fascial Plane Block (RIFPB) and catheter placement. (A) The red dotted arrows indicate the direction of catheter placement. (B) Probe placement along the costal margin near the sternum, with the probe oriented diagonally from the nipple toward the epigastric region at the T7 level. (C) Ultrasound image showing local anesthetic (LA) spread beneath the rectus abdominis muscle (RAM) at the T7 costal cartilage (CC) level. The red arrowheads indicate the needle trajectory.

Additionally, RIFPB was performed by first identifying the space between the rectus abdominis muscle and T6-T7 costal cartilage. Taking advantage of the clear anatomical landmarks provided by the costal margin, we performed direct hydrodissection with 10 mL of 0.25% levobupivacaine to establish this plane bilaterally; catheters were inserted. Post-operatively, intermittent boluses of 10 mL of 0.25% levobupivacaine were administered through each catheter every 12 hours (Figure 3).

This standard 12-hour interval proved sufficient for maintaining consistent pain control throughout the recovery period without the need for additional interventions or dose adjustments. The patient demonstrated excellent pain control in both the sternal and drain site regions from the immediate post-operative period (POD0). Post-procedure cold testing (using an ice cube) confirmed sensory blockade of the anterior cutaneous branches of the intercostal nerves from T2 to T8 bilaterally, encompassing both the sternal (T2-T6) and epigastric drain site (T7-T8) regions. The following morning, repeat cold testing revealed that sensory blockade in T2-T6 dermatomes had resolved, while blockade in the T7-T8 regions persisted, demonstrating the differential duration of the SPIPB and RIFPB techniques.

The excellent pain control continued through POD4. Pain scores remained consistently low and stable (NRS score 0-2 at rest, 2 with coughing) throughout this entire period with the scheduled bilateral catheter boluses administered every 12 hours. No breakthrough pain was observed, and notably, beyond the standard intraoperative fentanyl and acetaminophen administered at sternal closure, no additional systemic analgesics were required at any point during the post-operative course, despite the availability of our tiered analgesic protocol that included oral tramadol/acetaminophen and rescue IV-PCA.

Despite the patient's compromised cardiac function (ejection fraction 33%), early mobilization was successfully achieved with the patient able to sit up in bed on POD1 without pain limitations. The patient continued to progress with mobilization until POD4, when the chest drains were removed. Following drain removal on POD4, both RIFPB catheters were also removed, as they had successfully fulfilled their purpose of providing consistent analgesia throughout the critical early mobilization period. After RIFPB catheter removal, the patient continued to report minimal pain (NRS 0-1) and did not require any additional analgesics. No sharp or rebound pain was observed during the transition from catheter-based analgesia to recovery without regional techniques.

## Discussion

In Case 1, while the DPIPB catheters provided excellent sternal pain control as confirmed by cold testing showing sensory blockade in T3-T6 dermatomes, they failed to provide effective analgesia in the epigastric drain site region. Despite administering additional local anesthetic boluses through the DPIPB catheters, cold testing confirmed that there was no extension of the sensory blockade to the T7-T8 dermatomes where the drains were located. This objectively demonstrates the limitations in DPIPB's caudal spread. Adequate pain control in the epigastric region was ultimately achieved through the addition of oral analgesics rather than through the extension of the regional block.

In contrast, the combination approach used in Case 2, with SPIPB for sternal coverage and RIFPB catheters specifically targeting the epigastric region, provided comprehensive analgesia without any breakthrough pain, despite SPIPB's theoretical limitations in providing sustained analgesia for the upper sternal area. Two key differences in our approach between cases should be highlighted: first, the anatomical targets differed, with DPIPB targeting a deeper plane while the SPIPB/RIFPB combination addressing both superficial parasternal and rectus sheath regions; second, the catheter placement strategy differed, with DPIPB catheters placed at the T4-T5 level in Case 1 versus a combination of single-shot SPIPB with strategically placed RIFPB catheters at the T6-T7 costal margin in Case 2. This difference in approach appears critical, as it enabled precise targeting of both pain sources-sternal incision and drain sites-in the second case. Most notably, the patient in Case 2 required no systemic analgesics beyond the standard intraoperative fentanyl and acetaminophen administered at sternal closure-no oral analgesics, no rescue opioids, and no IV-PCA were needed throughout the recovery period.

From a technical perspective, these fascial plane blocks present different levels of procedural complexity and safety considerations. The SPIPB technique involves needle advancement to a relatively superficial plane above the ribs, while the RIFPB targets the costal margin as a clear anatomical landmark. In contrast, the DPIPB requires deeper needle placement to access the transversus thoracis muscle, which is typically very thin and lies in close proximity to the pleura, making this technique potentially more challenging and requiring greater caution to avoid pleural puncture. These technical differences may influence the selection of appropriate techniques based on anesthesiologist experience and individual patient factors.

Clinically, we have observed that many post-sternotomy patients complain more about epigastric drain site pain than sternal incision pain, with considerable individual variation. Extubation timing also varies based on preoperative status and intraoperative factors, further complicating pain management. Targeted regional anesthetic catheters help manage this variability by providing localized, anatomically specific analgesia during the critical transition from intubation to mobilization. This approach is particularly valuable for patients with compromised cardiac function, where opioid-sparing techniques facilitate early mobilization and reduce respiratory complications.

The rectus sheath block (RSB) represents another promising option for managing epigastric region pain, as demonstrated in a recent randomized controlled trial [7]. This study showed that adding RSB to parasternal blocks significantly improved pain control and respiratory performance while reducing opioid consumption after cardiac surgery. Both RSB and RIFPB are effective techniques targeting the anterior cutaneous branches of the intercostal nerves T6-T8, which provide sensory innervation to the epigastric region where drains are typically placed following cardiac surgery. They achieve this via different anatomical approaches, with each technique accessing these nerve branches at distinct anatomical locations. The RIFPB approach used in Case 2 targets these nerves at a different anatomical location compared to RSB. Our technique of placing RIFPB catheters at the costal margin, while not entirely novel but rather a refinement of existing approaches specifically for cardiac surgical patients, offers important advantages: it eliminates the risk of peritoneal puncture by using the costal arch as a clear anatomical landmark and provides a more proximal nerve block compared to RSB. This proximal approach may provide a more stable neural blockade when faced with anatomical tissue disruption caused by drain insertion.

The catheter placement technique we describe offers simplicity, stability, and safety for extended analgesia. While RIFPB has been previously described as a single-shot technique [8], our case demonstrates the feasibility and potential benefits of catheter placement for extended analgesia through intermittent bolus administration. However, we do not necessarily advocate for a four-catheter approach (bilateral sternal plus bilateral epigastric) as standard practice. Our experience suggests that a single-shot technique may be sufficient for the sternal component in many patients, with catheters reserved for the epigastric region where pain often persists longer due to drain irritation. This selective catheter placement strategy balances comprehensive analgesia with procedural efficiency and resource utilization. The comparative efficacy of catheter-based RSB versus RIFPB for post-operative cardiac surgical pain management warrants further investigation, particularly regarding ease of catheter placement, stability, and optimal administration regimens.

Recent comparative studies provide additional context for our findings. Yadav et al. [9] conducted a randomized controlled trial comparing DPIPB and SPIPB (termed parasternal intercostal nerve block (PICNB) in their study) in 60 adult cardiac surgical patients. Both techniques significantly reduced post-operative opioid consumption compared to a control group, but the differences between the two blocks themselves were not statistically significant, suggesting that either technique can be effective.

In contrast, Mansour et al. [10] found that SPIPB provided a longer time before the first request for rescue analgesia (7.8 ± 1.7 hours vs. 6.7 ± 1.4 hours for DPIPB) and lower overall morphine usage in the first 24 hours post-operation (4.8 ± 1.0 mg vs. 7.8 ± 2.0 mg), indicating potentially greater efficacy with SPIPB. These findings are interesting in the context of Case 2, where we observed excellent analgesia without supplemental oral analgesics. However, we must emphasize that this outcome cannot be attributed to SPIPB alone, as it was used in combination with RIFPB catheters targeting the epigastric region. This combined approach specifically addressed both pain sources simultaneously, which likely explains the comprehensive analgesia achieved. Interestingly, despite negative cold tests in the T2-T6 dermatomes by POD1 morning, the patient continued to report minimal pain in the sternal region, suggesting that the analgesic effect of SPIPB may persist beyond the duration of cutaneous sensory blockade detectable by cold testing.

Recent studies further contextualize our findings on block distribution and duration. Zhang et al. [11] found that DPIPB in 113 cardiac surgery patients provided reliable T4-T6 sensory blockade with less consistent coverage in T2-T3 dermatomes, limited effectiveness in T7, and a mean duration of 17 hours. Similarly, Chen et al. [12] reported that preemptive DPIPB offered effective immediate post-operative analgesia but inadequate coverage beyond 24 hours. These findings align with our Case 1 observations-excellent initial sternal analgesia with T3-T6 coverage that required reapplication by the following morning and lack of coverage in T7-T8 dermatomes where drains were located. This evidence supports our catheter-based approach to extend analgesia beyond the natural block duration and explains the breakthrough epigastric pain observed in Case 1.

The value of catheter-based approaches is further supported by Zhan et al. [13], who demonstrated that bilateral continuous DPIPB blocks accelerated recovery after open heart valve replacement surgery. Their study of 110 patients showed that continuous DPIPB blocks resulted in significantly lower pain scores at rest and during exercise, shorter times to extubation and mobilization, and reduced lengths of stay in both the ICU and hospital compared to controls.

While previous studies have focused primarily on continuous infusion through catheters, our approach differs in that we utilized intermittent scheduled boluses specifically timed before rehabilitation activities. Our standardized 12-hour interval for bolus administration was based on institutional protocols designed to align with nursing shifts and rehabilitation schedules. However, we recognize that a more individualized approach with bolus administration triggered by pain emergence rather than fixed intervals might offer advantages in some patients. The optimal timing for bolus administration through these catheters warrants further investigation to balance predictable analgesia with personalized pain management. This strategic timing of bolus administration represents a novel aspect of our technique, allowing for optimal analgesia during the most painful periods of mobilization while potentially reducing the total local anesthetic dose compared to continuous infusion. The targeted administration before rehabilitation activities may contribute to improved patient comfort during early mobilization, potentially facilitating more effective participation in recovery exercises. This approach combines the extended analgesia benefits of catheter placement with the flexibility and safety advantages of intermittent administration, representing a refinement in technique that warrants further investigation in larger studies.

As highlighted in a recent practice advisory by the Society of Cardiovascular Anesthesiologists [1], moderate to severe pain after cardiac surgery is relatively common and increases the risk of post-operative cardiopulmonary complications while delaying hospital discharge. While opioids have traditionally been used for post-cardiac surgery pain control, they are associated with serious adverse effects that can be particularly problematic in this patient population. The advent of fascial plane blocks has significantly expanded regional analgesia options for cardiac surgical patients-an approach that was previously limited due to concerns about neurological complications in the setting of systemic anticoagulation. Our case experiences align with this advisory's emphasis on multimodal analgesia to decrease reliance on opioids, reduce opioid-related adverse effects, and promote early recovery after cardiac surgery.

We selected levobupivacaine for its favorable safety profile, particularly its reduced cardiotoxicity compared to bupivacaine [6]. The total dose of levobupivacaine administered on the initial day was 100 mg (1.43 mg/kg) in Case 1 (40 mL of 0.25% solution for bilateral DPIPB) and 150 mg (1.97 mg/kg) in Case 2 (40 mL for bilateral SPIPB plus 20 mL for bilateral RIFPB). For subsequent days, the maintenance dose was standardized at 50 mg per bolus (20 mL of 0.25% solution), administered twice daily for a total daily dose of 100 mg in both cases. All doses remained well below the recommended maximum safe dose of 2.5-3 mg/kg. Despite levobupivacaine's improved safety profile, a careful technique to avoid intravascular injection remains essential. Our intermittent bolus approach, rather than continuous infusion, balances extended analgesia with safety, allowing assessment between doses while maintaining effective pain control. Additionally, intermittent bolus administration likely provides a more extensive spread of local anesthetic within the fascial plane compared to continuous infusion. The higher injection pressure achieved during bolus delivery can overcome tissue resistance and create a wider distribution of the anesthetic agent, potentially improving block quality and coverage. This mechanical advantage of bolus administration may be particularly relevant in fascial plane blocks where adequate spread is essential for covering multiple nerve branches across several dermatomes. This strategy is particularly valuable during the vulnerable early post-operative period when cardiovascular stability may be compromised.

Several limitations should be acknowledged. As a two-case report, our findings cannot determine which approach is definitively superior, and individual patient factors may influence the optimal choice of technique. The total local anesthetic doses used in both approaches remained within safe limits, but systematic studies are needed to establish optimal dosing regimens for these catheter-based techniques. Additionally, the specific contribution of each block in our combined approach cannot be precisely determined without controlled comparative studies.

## Conclusions

Catheter-based regional anesthetic techniques offer promising solutions for comprehensive pain management after cardiac sternotomy, facilitating early mobilization and reducing systemic analgesic requirements. Both DPIPB catheters and the combined SPIPB/RIFPB approach demonstrated efficacy in our cases, each with its own clinical characteristics. Our catheter placement technique for RIFPB at the costal margin represents a novel approach that offers anatomical clarity, procedural safety, and targeted analgesia for epigastric drain site pain-an often overlooked but a significant source of discomfort after cardiac surgery. Additionally, our approach of administering intermittent boluses timed specifically before rehabilitation activities optimizes analgesia during the most challenging periods of mobilization while balancing safety considerations. This strategic timing represents a meaningful refinement over continuous infusion or as-needed boluses. Further research in larger populations is needed to establish optimal strategies for various cardiac procedures, particularly regarding catheter placement techniques and bolus timing in relation to rehabilitation activities.
